# Feasibility and acceptability of remote APOE-genotyping among research volunteers of an online recruitment registry (The Dutch Brain Research Registry)

**DOI:** 10.1016/j.tjpad.2025.100099

**Published:** 2025-02-24

**Authors:** L. Waterink, S.J. van der Lee, D. Nijland, F.I. van der Zee, L.N.C. Visser, Y.A.L. Pijnenburg, S.A.M. Sikkes, W.M. van der Flier, M.D. Zwan

**Affiliations:** a Alzheimer Center Amsterdam, Neurology, Vrije Universiteit Amsterdam, Amsterdam UMC location VUmc, Amsterdam, the Netherlands; b Amsterdam Neuroscience, Neurodegeneration, Amsterdam, the Netherlands; c Genomics of Neurodegenerative Diseases and Aging, Human Genetics, Vrije Universiteit Amsterdam, Amsterdam UMC location VUmc, Amsterdam, the Netherlands; d Department of Medical Psychology, Amsterdam UMC location AMC, University of Amsterdam, Amsterdam, the Netherlands; e Amsterdam Public Health research Institute, Quality of Care, Amsterdam, the Netherlands; f Faculty of Behavioural and Movement Sciences, Department of Clinical, Neuro and Developmental Psychology, Vrije Universiteit, Amsterdam, the Netherlands; g Department of Epidemiology and Data Science, Amsterdam University Medical Center, Vrije Universiteit Amsterdam, Amsterdam, the Netherlands

**Keywords:** Registries, Recruitment, Pre-screening, Genetic risk, APOE, Alzheimer's disease

## Abstract

**Background:**

Participant recruitment for preclinical Alzheimer's disease (AD) prevention studies is challenging. Online registries facilitate large scale prescreening of individuals at risk for AD to accelerate recruitment. *APOE*-prescreening has the potential to better identify at-risk individuals. This study investigated the feasibility and acceptability of at-home *APOE*-genotyping in cognitively-normal registrants of an online registry.

**Methods:**

We invited 9,287 cognitively-normal registrants of Dutch Brain Research Registry (DBRR) aged 50 to 75 for at-home *APOE*-genotype testing, without receiving the results. Feasibility was measured by participation ratio (participation/interested), swab-return ratio (returned-swabs/participation), and genotyping-success ratio (analyzed swabs/returned swabs). Acceptability was measured with online questions about information provision and project scope. We explored prescreening questions potentially reducing screen-failures.

**Results:**

Feasibility was high with an 0.89 participation ratio (2,886/3,251), 0.90 swab-return ratio (2,886/2,597), 0.99 genotyping-success ratio (2,558/2,597). Acceptability was high, as participants were content with the information provision (87 %-97 %, *n=* 1,709–1,894), which was also well understood (91 %-93 %, *n* = 1,772–1,802). Among successful-analyzed swabs (*n* = 2,558), 27 % participants were *APOE*-ε4 heterozygote (*n* = 703), and 2 % homozygote (*n* = 60). Prescreening on a positive family history leads to a third reduction in the number of invitations needed to identify one *APOE*-ε4 carrier.

**Conclusion:**

Our results suggest that *APOE-*ɛ4 genotyping in participants of an online research registry is feasible, well received and could be used to prescreen individuals at risk for AD for prevention studies. Adding a positive family history before invitation for *APOE*-genotyping, would further improve the prescreening process and reduce screen failures when identifying carriers.

## 1 Introduction

Improving recruitment and screening methods to accelerate inclusion of participants is a priority in Alzheimer's disease (AD) clinical research [1]. The preclinical phase of AD before symptom onset provides a window of opportunity for intervention and the number of AD prevention studies is growing rapidly [2]. Identifying at-risk individuals and including eligible participants for prevention trials is challenging, as it requires large outreach and screening [3,4]. The average recruitment time for prevention trials is 2.9 years [2], and the screen failure rate is estimated at approximately 88 %, leading to a substantial waste of time and resources [5]. Online registries have the potential to efficiently accelerate recruitment because they host a vast pool of voluntary research participants, and can facilitate prescreening for multiple prevention studies based on demographic, health, and/or cognitive information to identify at-risk individuals and can invite screen failures for other studies [[6], [7], [8], [9], [10]].

After advanced age, family history is the second most significant risk factor, and it is estimated that genetic factors contribute to 70 % of AD cases [11]. In the general population, the most common genetic risk factor for AD is the apolipoprotein E ɛ4 variant (*APOE-*ɛ4) [[12], [13], [14], [15]]. Carrying one *APOE-*ɛ4 allele (heterozygote) increases the risk of developing Alzheimer's disease 3- to 4-fold, while having two *APOE-*ɛ4 alleles (homozygote) raises the risk by 10- to 15-fold [16]. *APOE-*ɛ4 carriership is therefore often used as inclusion or enrichment criteria in dementia research and clinical trials to increase the likelihood of including cognitively normal participants with AD pathology. In the general population, 20–30 % carry at least one copy of the APOE e4 allele, and only 2–3 % have two copies [12,15]. Consequently, identifying 25 APOE e4 carriers likely requires screening over 100 individuals, resulting in a screen failure rate of approximately 75 %. Most clinical trial sites lack the necessary funding, resources, outreach capabilities, and research staff to effectively recruit and screen for at risk participants, leading to delays in recruitment and enrolment [4,17]. To advance dementia research, effective recruitment strategies for genetically eligible individuals are needed [18].

Several population-based online recruitment registries have integrated genetic data for prescreening [19,20], successfully enhancing the efficiency and effectiveness of recruiting cognitively unimpaired APOE-ε4 carriers for prevention studies [21]. For instance, the Generation Studies, a pharmacological primary prevention trial, recruited 1626 cognitively unimpaired APOE ɛ4 carriers across 120 sites in 24 countries. In US alone, 4055 individuals were screened for which GeneMatch - an online recruitment registry - was the most successful recruitment strategy accounting for 49 % of study referrals [22,23]. GeneMatch invites a pool of research volunteers enriched for genetic risk, increasing the likelihood of identifying eligible participants. In this manner, recruitment registries like GeneMatch can efficiently and effectively accelerate enrollment in AD prevention studies. Currently, this prescreening approach has not been validated in registries outside the US.

In The Netherlands, The Dutch Brain Research Registry (DBRR; in Dutch: Hersenonderzoek.nl) provides support for recruitment of research participants, with prescreening based on a simple intake survey [23]. A prior survey among cognitively healthy DBRR participants revealed strong interest in genetic dementia research [24], though their acceptance of actual genetic testing for prescreening purposes is yet to be determined. Given the complexity of understanding AD genetic risk, assessing the acceptance of *APOE*-genotyping using buccal swabs and evaluating research volunteers’ comprehension of AD genetic risk are critical for the implementation of genetic prescreening strategies in an online registry. Currently, to prescreen for individuals at risk for cognitive decline the DBRR uses AD-related risk factors, such as family history of dementia and subjective memory complaints [[25], [26], [27], [28]]. Although a positive family history and subjective memory complaints are known to associated with AD risk [29], the potential of these factors to identify genetically at-risk individuals has not been studied. In this study we aimed to assess feasibility and acceptability of remote *APOE*-genotyping with buccal swabs in cognitively-normal individuals with the goal to implement genetic prescreening within the DBRR. Secondly, we explored which prescreening question (a positive family history and subjective memory complaints) performed best in identifying *APOE* ɛ4 carriership and would reduce number needed to screen. When successful, this can be implemented on larger scale and help dementia researchers recruit at-risk individuals more efficiently and effectively.

## 2 Methods

### 2.1 Dutch Brain Research Registry

The Dutch Brain Research Registry (DBRR; in Dutch: Hersenonderzoek.nl) is a nationwide online registry for participant recruitment for brain disease studies in The Netherlands [30]. The DBRR provides information for a lay audience on currently recruiting studies, study results, brain disease related topics and information on study participation to actively engage registrants. Registration in the DBRR is free and open to anyone from the age of 18. Upon subscription registrants fill out a basic questionnaire about personal-, health- and lifestyle information. Based on this information and study specific inclusion criteria, registrants are prescreened and invited to studies.

### 2.2 Buccal swab for APOE-genotyping

For buccal swab sampling we used the Isohelix Buccal Swabs with RapiDri pouch (Westburg, life sciences). For this study, 3000 swabs were available to send out for APOE-genotyping. Participants received a test-kit at home including cover letter, information letter, buccal swab, detailed collection instructions, return envelope and information to optionally donate genetic material for a Biobank. Participants are instructed not to eat or drink anything one hour prior to swabbing the inside of their cheek with the buccal swab. For quality assurance, all samples include sex verification, which is cross-checked with the information provided at intake and on the laboratory requisition form. APOE haplotypes were determined from direct genotyping of two single nucleotide polymorphisms (SNPs): rs429358 (hg19, chr19:45,411,941, T > C) and rs7412 (hg19, chr19:45,412,079, C > T). Both SNPs were measured in 384 wells PCR plates using an allelic discrimination assay of ThermoFisher (Waltham, USA). Each well contained 2 ng DNA, 1x Type-it Fast SNP PCR Mix (Qiagen, Hilden, Germany) and 1x Taqman assay (ThermoFisher) in a reaction volume of 2ul. Amplication was done in a PCR machine with recommended cycling conditions and end point analysis was done in a 7900HT Real time PCR system (ThermoFisher) [31]. Remaining genetic material was stored in the DBRR Biobank for future research if consent was given, or otherwise destroyed after *APOE* genotyping.

### 2.3 Participant selection and informed consent procedure

Registrants between 50 and 75 years old without a self-reported diagnosis of dementia or mild cognitive impairment (MCI) were invited from the DBRR. With purposeful sampling we aimed for a heterogeneous sample regarding gender and education by first inviting individuals that are underrepresented in the DBRR (i.e. men and individuals with less educational attainment). As part of the intake questionnaire, participants report on their education level, family history of dementia and presence of subjective memory complaints. Education was categorized into (1) vocational education or less (equivalent of ≤ 12 years of education) and (2) higher vocational or academic education (equivalent of ≥13 years of education). Subjective memory complaints were assessed with the questions ‘Do you have memory problems?’ and ‘Are you concerned about your memory problems’.

Based on 3000 available buccal swabs for this study, we invited 9287 registrants. The design of the study was to not disclose results to participants. Interested registrants were provided with more online information, including both an information video about genetic risk for AD and het project scope, and written participant information. Participants were informed that results of *APOE*-genotyping were used to prescreen them for future research, and that they might learn their *APOE*-genotype when participating in future studies but only if they consent to participate and disclosure at that time. After this extensive information they had the option to provide online informed consent. Registrants that were not interested in participation or declined informed consent (i.e. non-participants) were asked about their reasons in an open text field.

### 2.4 Surveys

Two short surveys were administered, one immediately after participants provided informed consent (prior to genotyping) and the other after the swab kits were sent to participants' homes. The first survey was based on the education module of GeneMatch [22], and consisted of questions about 1) comprehension about project scope and genetic risk for dementia, 2) acceptability of our information provision, 3) motivation for participation, and 4) self-estimated risk for dementia. To make sure we used most recent information on subjective memory complaints and family history of dementia, these questions were repeated in the survey. In part 1 of the survey, when participants answered the questions incorrectly, they were immediately provided with the right answers to ensure they were well-informed. If participants indicated the information provided was insufficient, we included a follow-up open-ended question asking how to improve our information provision in an open text field. In part 4, we asked participants to estimate their dementia lifetime risk (0–100 %), and their risk in the coming 5 years (0–100 %). Next, participants were given more information about dementia risk in the general population at 85 years of age, and were asked if they estimated their risk to be higher or lower compared to general population on 5-point Likert scale and their reasons. After participants returned the swab (or not), the second survey was administered. The survey included questions about the 1) acceptability of the buccal swab instructions or reasons for not returning the swab, and 2) the participants' understanding of the project scope, which was assessed by repeating two questions from the first survey part 1. The surveys were not mandatory, however participants were encouraged and reminded via email.

### 2.5 Data analysis

Feasibility was measured in response ratio (interested/invited), participation ratio (participated/interested), swab-return ratio (returned swabs/enrolled), genotyping-success ratio (analyzed swabs/returned swabs) and carrier-ratio (*APOE* ε4 carriers/analyzed swabs). Acceptability was described as the distribution of percentages of participants’ response to the different questions about contentment with information provision prior genotyping, comprehension of the project scope, understanding of buccal swab instructions, and motivation for study participation. Additionally, we coded reasons for declining study participation (open-ended question, coded by LW with MAXQDA), and subsequently described the percentage of the participants in each group. Differences in demographic characteristics were tested between participants and non-participants using one-way analysis of variance, Kruskal-Wallis tests, and Chi-square tests of proportions. Demographic characteristics, as well as differences in prescreening questions were tested between *APOE*-genotypes (non-carriers, heterozygotes and homozygotes), using one-way analysis of variance, Kruskal-Wallis tests, and Chi-square tests of proportions, with post hoc analyses where appropriate. To identify prescreening questions associated with *APOE*-ε4 carriership (non-carriers, heterozygotes and homozygotes), we performed a multivariable ordinal regression model (adjusted for sex and age). All statistical analyses were conducted using RStudio (version 4.3.2), *p*-level of 0.05 was considered statistically significant.

## 3 Results

### 3.1 Participant characteristics

Participants had a mean age of 67.7 (SD 6.8) years, 57 % (*n* = 1639) were female, 84 % (*n* = 2410) had a higher vocational or academic education, 19 % (*n* = 495) reported having subjective memory complaints, and 46 % (*n* = 1332) had a first-degree relative with dementia (Table 1). Among successful-analyzed samples *(n* *=* 2558), 27 % participants were *APOE*-ε4 heterozygote (ε3ε4 *n* = 640, ε2ε4 *n* = 63), 2 % homozygote (ε4ε4 *n* = 60), and 12 % carried the protective ε2 allele (ε2ε2 *n* *=* 14*,* ε2ε3 *n* = 302; Table 1). Most participants (98 %, *n* = 1913) did not have prior information about their genetic risk for AD. Participants estimated their lifetime risk on 50 % (IQR 25 %, 70 %), and within 5 years on 18 % (IQR 5 %, 45 %). After providing information about dementia risk (i.e. 15 % risk at the age of 85 in the general population), 19 % (*n* = 537) participants estimated their risk to be (much) higher compared to the general population. Endorsed reasons for being at higher risk were having a first-degree relative with dementia (88 %; *n* = 473), having memory problems (27 %; *n* = 145), and having health problems like cardiovascular or diabetes (17 %; *n* = 89, Supplementary Table 1).

**Table 1:** Demographics of participants.

	**Participants** (*n* = 2886)
**Age** (mean ±SD)	67.7 ± 6.8
**Sex,** (%)	
Male	1242 (43)
Female	1639 (57)
**Education**, n (%)	
Vocational or less	470 (16)
Higher vocational or academic	2410 (84)
**Subjective memory complaints,***n* (%)	494 (17)
**Relative with dementia,***n* (%)	
Yes	1331 (46)
No	1413 (49)
I don't know	134 (5)
**Percentage self-estimated lifetime risk,** (median [IQR])	50 [25,70]
**Percentage self-estimated risk in 5 years,** (median [IQR])	18 [5,45]
**Self-reported risk compared to general population,***n* (%)	
(Much) smaller	494 (17)
Similar	896 (31)
(Much) higher	537 (19)
**Prior genetic testing,***n* (%)	
No	1913 (66)
Yes, unpaid	33 (1)
Yes, paid	14 (<1)
***APOE* genotype,***n* / N (%)	
ε2/ε2	14 / 2558 (1)
ε2/ε3	302 / 2558 (12)
ε3/ε3	1479 / 2558 (58)
ε2/ε4	63 / 2558 (2)
ε3/ε4	640 / 2558 (25)
ε4/ε4	60 / 2558 (2)

Note: *SD* standard deviation *IQR* interquartile range *APOE* Apolipoprotein E. Percentages are based on total number participants that provided informed consent, or when indicated with *n* / N as percentage of total number with data available. Number missing for sex *n* = 5; education *n* = 6; subjective memory complaints *n* = 251; relative with dementia *n* = 8; self-reported risk compared to general population *n* = 959; prior genetic testing *n* = 926; percentage self-estimated lifetime risk *n* = 963; percentage self-estimated risk in 5 years *n* = 961.

### 3.2 Feasibility

**Fig. 1:**
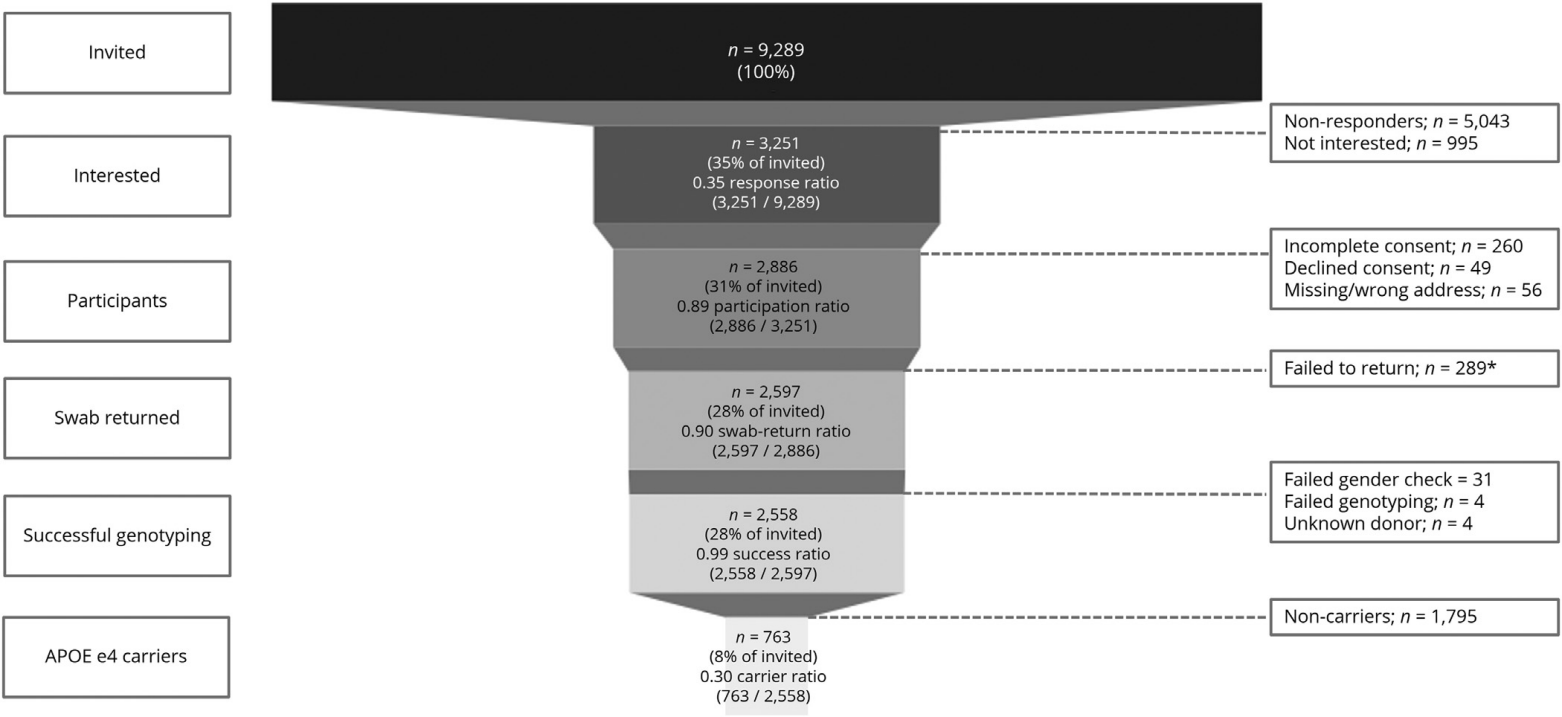
Funnel displaying participant enrollment, successful genotyping and *APOE* ɛ4 carriers. *Most participants indicated wanting to complete their participation.

Fig. 1 shows the complete participant flow from invitation to *APOE*-genotyping. Of 9289 total invited registrants, 3251 registrants were interested (0.35 response ratio), of which 2886 participated and were sent a buccal swab to their homes (0.89 participation ratio). Of the participants, 2597 returned the buccal swab (0.90 swab-return ratio) of which 2581 were successfully genotyped (0.99 genotyping-success ratio). We identified a total of 763 *APOE-*ε4 carriers (0.30 carrier ratio). Based on 9289 invited registrants, 8 % were carriers, meaning that identifying one APOE-ε4 carrier required inviting twelve registrants from the DBRR of which at least four participants were genetically tested.

Our material loss was 4 % (2886 participants / 3000 available swabs), primarily due to swabs getting lost during postal transit requiring replacement kits, and some participants mistakenly receiving duplicate swabs due to double invitations. Thirty-one samples failed sex verification, additional four samples failed genotyping and four samples had an unknown donor due to duplicate or missing identification numbers. 1976 participants (76 %) consented to donate remaining DNA material to the Biobank.

In line with General Data Protection Regulation (GDPR), genetic results were shared with 13 participants (1 %) upon request of whom five were heterozygotes for *APOE* ε4 and one homozygote for *APOE* ε4. Carriers were not surprised with their results, mostly due to family history of dementia, and therefore expected to have elevated risk compared to the general population. After our phone call and receiving the information letter, these registrants did not reach out for a follow-up consult.

### 3.3 Acceptability

**Fig. 2:**
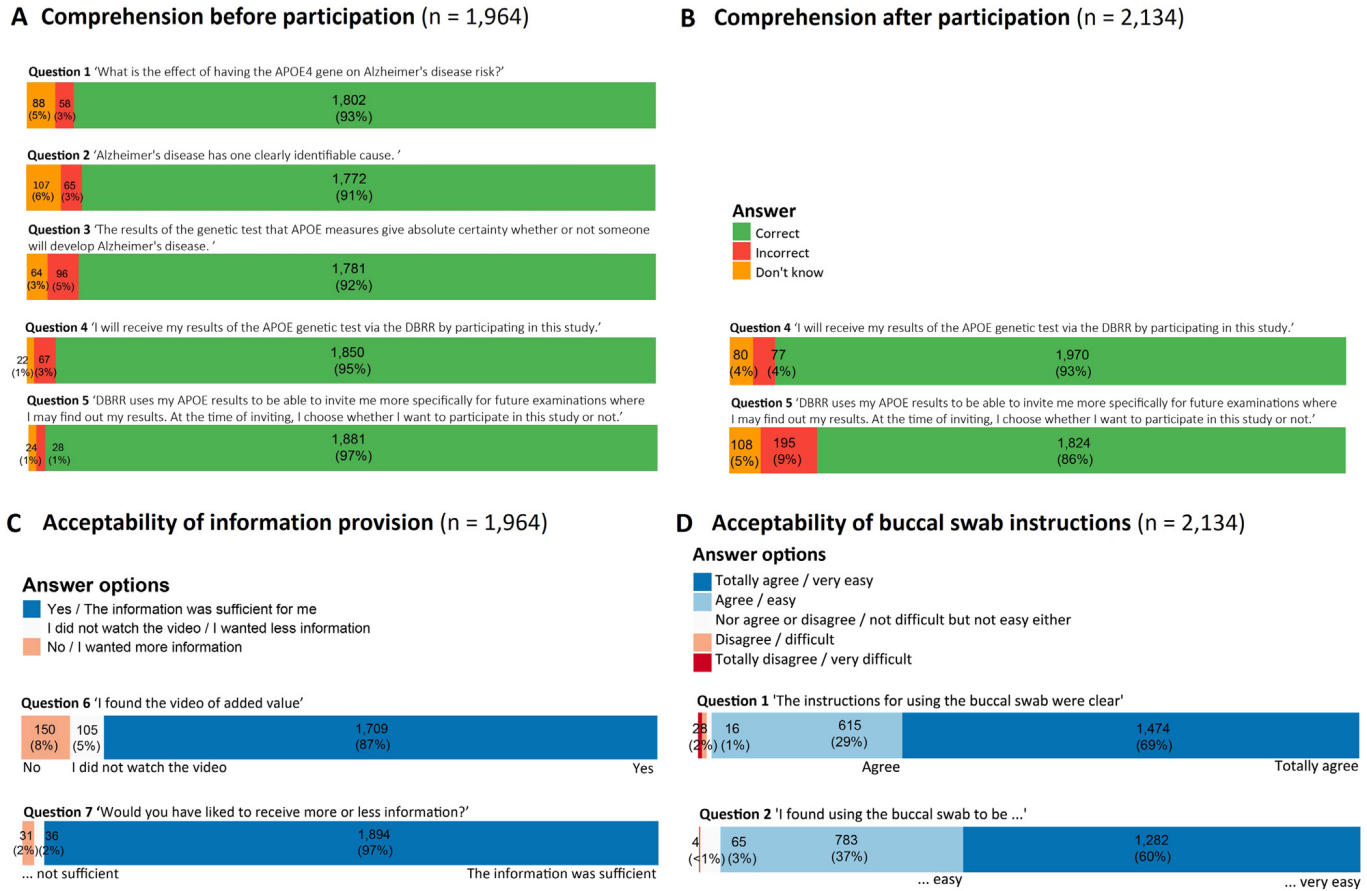
Results on the acceptability surveys. A) comprehension about AD risk and the study scope before participation; B) comprehension about study scope after participation; C) acceptability of the information provision before participation; D) acceptability of buccal swab instructions after participation.

Among participants, 1964 (68 %) filled out the survey before returning the swab, and 2126 (74 %) filled out the survey after returning the swab. Fig. 2 shows results of both acceptability surveys. Information about genetic risk for dementia was well understood (Fig. 2A Question 1 to 3: 91 % - 93 %, *n* = 1772 - 1802). At start of the study comprehension about the project scope (i.e., not disclosing APOE results; and use of APOE results for prescreening for future studies) was high (Fig. 2A Question 4 and 5: 95 % - 97 %, *n* = 1850–1881), although this percentage slightly dopped after returning the swab (Fig. 2B Question 4 and 5: 86 % - 93 %, *n* = 1824–1970). Almost all participants found the information provision sufficient (Fig. 2C Question 6: 97 %; *n* = 1895), and the information video to be of added value (Fig. 2C Question 7: 87 %; *n* = 1709). Participants indicating information provision was insufficient (2 %, *n* = 31) mentioned considering to provide more explanation about the reason for not disclosing results, the broader research context, and if invitation of future research indicates they are at-risk for dementia and what the future studies entails. The instructions for the buccal swab were considered clear (Fig. 2D Question 1: 97 %, *n* = 2089), and using the swab (very) easy (Fig. 2D Question 2: 98 %, *n* = 2065). As reasons to participate, most participants endorsed ‘to contribute to scientific research’ (97 %; *n* = 1867), and one-third (34 %; *n* = 656) endorsed ‘to participate in future research to learn my genetic risk of dementia’ (Table 2). Among participants that did not return their swab, 59 participants provided reasons which were mostly time related or simply forgot (50 %, *n* = 32), health related (15 %, *n* = 9), and 18 % (*n* = 11) indicated they never received their swab at home. Most of these participants wanted to complete their participation, and were sent a replacement kit.

**Table 2:** Number of participants endorsing reasons to either participate or decline invitation.

	Endorsed, *n* (%)
**Reasons for participation** (N = 1964)	

I want to contribute to scientific research	1866 (97)
I want to participate in future research where I will learn my risk of dementia	656 (34)
Participating in this research has no personal consequences for me at this time	401 (21)
I don't want to know my genetic risk for dementia	12 (1)

**Reasons to decline invitation** (N = 344)	

Time related	104 (30)
Personal health issues	61 (18)
Wanting to know the results of genetic test	59 (17)
Other personal reasons	55 (16)
Privacy concerns	30 (9)
I don't want to know my genetic risk for dementia	26 (8)

Notes. Results are presented as the number of participants *n* (%) out of the total number participants (N) that responded to the survey. Reasons for participation were multiple choice, and reasons to decline invitation were responses to open-ended questions.

### 3.4 Reasons for non-participation

Of 9289 total invited, 6403 (69 %) registrants did not respond, were not interested in participation, declined informed consent or (pre)screen failed (i.e. non-participants). Supplementary Table 2 shows the demographics of non-participants. Compared to participants, non-participants were slightly younger, more often had a vocational education or less, more often reported subjective memory complaints but less often had a first-degree family member with dementia (Supplementary Table 2). Among 995 registrants that declined participation, 344 registrants (35 %) provided reasons which were mostly time related (30 %; *n* = 104), health related (18 %; *n* = 61), or wanting to receive results of *APOE* genotyping (17 %; *n* = 59; Table 2). Only 8 % (*n* = 26) replied not wanting to learn their genetic risk for dementia, and 9 % (*n* = 30) mentioned privacy concerns (Table 2).

### 3.5 Prescreening questions to identify APOE ε4 carriers

Compared to non-carriers, hetero- and homozygotes carriers were younger, more often had a first degree relative with dementia, estimated their lifetime risk higher (on a scale to 0–100 %), and reported their dementia risk was (much) higher compared to the general population (Table 3). Results of multivariable ordinal regression analysis showed that a positive family history of dementia was significantly associated with *APOE*-ε4 carriership, with individuals reporting a family history being more than twice as likely to carry an additional copy of the *APOE*-ε4 allele compared to those without a family history (OR = 2.14, 95 %CI = 1.66 – 2.77; Supplementary Table 3). In other words, a positive family history of dementia as a prescreening question prior to *APOE*-genotyping would reduce the number needed to invite by a third (corrected for response-, participation-, swab-return and genotyping success ratio, as shown in Fig. 1). To identify a one *APOE*-ε4 carrier, this prescreening approach would require inviting eight individuals and analyzing two swabs, compared to twelve invitations and four swabs needed for the unscreened registry (Supplementary Figure 1). To identify one *APOE*-ε4 homozygote, prescreening with a positive family history for dementia would reduce the number of invitations (155 vs. 32) and swabs needed (48 vs. 10) by 80 %).

**Table 3:** Demographics and prescreening questions stratified by genotype.

	**Non-carriers** (n = 1812)	**Heterozygotes** (n = 703)	**Homozygotes** (n = 60)	**p-value**
**Age** (mean ±SD)	68.1 (6.7)^a^	67.3 (6.7)	65.6 (7.3)	0.001
**Sex,** n (%)				
Male	786 (43)	293 (42)	23 (38)	0.482
Female	1006 (56)	408 (58)	37 (62)	
**Subjective memory complaints,** n (%)	296 (16)▼	136 (19)	17 (28)	0.030
**Relative with dementia,** n (%)				
Yes	745 (41)▼	416 (59)▲	42 (70)▲	<0.001
No	959 (53)▲	257 (37)▼	16 (27)▼	
I don't know / Prefer not to say*	87 (5)	28 (4)	2 (3)	
**Percentage self-estimated lifetime risk** (median [IQR])	50 [25,69]	50 [25,70]^b^	60 [50,77]^a^	0.001
**Percentage self-estimated risk in 5 years** (median [IQR])	15 [5,40]	20 [6,50]^b^	25 [8,50]	0.033
**Self-reported risk compared to general population,** n (%)				
(much) smaller	342 (19)▲	98 (14)▼	5 (8)	<0.001
Similar	569 (31)▲	199 (28)▼	14 (23)	
(much) higher	291 (16)▼	178 (25)▲	19 (32)▲	

Notes. Differences were tested using one-way analysis of variance, Kruskal-Wallis, Chi-square test including post-hoc analysis. *Excluded from chi-square and post-hoc analysis. Post-hoc analysis showed significant difference with:

a higher compared to other groups.

b higher compared to non-carriers.

▲ more often reported (positively associated);^▼^less often reported (negatively associated). Number missing for age *n* = 5; sex *n* = 5; subjective memory complaints *n* = 220; relative with dementia *n* = 7; self-reported risk compared to general population *n* = 852.

## 4 Discussion

The main findings of this study were that *APOE-*ɛ4 genotyping for an online recruitment registry using at-home test kits is feasible, reflected in high participation-, swab-return and genotyping-success ratios. Moreover, utilizing *APOE*-genotypes as a prescreening tool for future research is well-accepted by cognitively healthy volunteers, supported by the positive responses on survey on information provision, understanding of genetic dementia risk and project scope. Adding a positive family history before invitation for *APOE*-genotyping would further improve the prescreening process and identify *APOE*-ε4 carriers more readily.

*APOE*-genotyping among cognitively normal research volunteers enlisted in an online recruitment registry identified 25 % heterozygous and 2 % homozygous *APOE*-ε4 carriers, which aligns with the expected frequencies in the general population [12,15].We had anticipated a higher enrichment of *APOE*-ε4 carriers given the relatively high proportion of participants with a family history of dementia. A positive family history of dementia was strongly associated with *APOE*-e4 carriership, with individuals reporting a family history being more than twice as likely to carry an additional copy of the APOE ε4 allele compared to those without a family history. Carriers justly tended to estimate their dementia risk higher than non-carriers, and often endorsed a family history of dementia as the reason for this perception. In line with this finding, the small number of participants carrying an ε4-allele who requested their results were unsurprised by their heightened risk, due to AD ‘running in their family’. These findings emphasize the influence of family history on perceived and actual genetic risk, while underscoring its importance in shaping individuals' understanding and responses to genetic information - potentially even more so for those without a family history of dementia. Using self-reported family history as an inclusion criterion can also introduce limitations as some individuals with preclinical AD report no family history of AD. For example, in the A4 study of solanezumab for preclinical AD, about 25 % of amyloid-positive participants reported no family history of AD [32]. Therefore, prescreening for other risk factors might be important to capture the full spectrum of individuals with preclinical AD.

Previous studies have shown *APOE* result disclosure is safe among cognitively healthy individuals [[33], [34], [35], [36], [37], [38]]. In the context of an online registry, however, disclosure is complex since genetic counseling to ensure accurate interpretation of results is time-consumable and therefore limits scalability. Comparable to other recruitment registries, the DBRR did not share results of *APOE*-genotyping with participants [19,22]. In The Netherlands, sharing research results with participants is not legally required, but the General Data Protection Regulation (GDPR) grants individuals the right to access their personal data, which includes “the right to know” what data are being processed and how are they used. Therefore, participants who actively requested their genotyping results were provided with them. Participants were contacted by a medical doctor by phone for disclosure and received their results by mail, along with a brochure from Alzheimer Netherlands titled "Maintain Your Brain Health". The disclosure process and materials were reviewed by a genetic counselor. Only a small number requested their results (*n* = 13; 1 %), and from the total invited only 59 registry volunteers (that provide a reason) declined study participation was because of non-disclosure of results. Thus, our results support previous findings that the lack of genetic disclosure does not constitute a barrier to provide genetic material for prescreen purposes [19,22]. Moreover, the survey results showed that only one-third (34 %) participated because they wanted to learn their dementia risk by participating in future research. These results underscores that the majority are predominantly motivated by altruistic reasons to participate and donate genetic material for prescreening purposes. On the other hand for some, sharing results can increase participant motivation and engagement in research [24,39,40]. Allowing participants to choose whether they want to receive their results seems to be the most suitable approach for the acceptance of genetic testing. Future studies are needed to evaluate the safety of scalable disclosure protocols in the context of a recruitment registry.

Results of the surveys showed that acceptability of genetic tests for prescreening among cognitively older adults enrolled in an online registry was high which also reiterated in high participation ratio (number participating / number interested 0.89). Other recruitment registries have demonstrated comparable success, for instance the Brain Health Registry's GenePool showed high acceptance and high participation ratio (0.93) [19]. The DBRR employs a stepwise consent procedure for online studies by first sending a short invitation email, secondly providing condensed research information after which participants express interest, followed by the official informed consent letter approved by the Ethics Committee. Unique for this study was the use of an educational video, which was appreciated by participants complementary to the text which might have influenced participation ratio positively. Registrants with fewer years of education less often participated (i.e. did not respond, not interested, incomplete or declined informed consent or failed to return swab), which suggests that information provision can be optimized especially for individuals with lower educational attainment. A few participants mentioned considering to provide more explanation about the reason for not disclosing results, the broader research context, if invitations for prevention studies indicate heightened risk for dementia and what the future studies entail. Moreover, informed consent procedure might be unclear to some, possibly due to the lengthy information which has been described as a barrier for research participation [41]. A previous study conducted among DBRR registrants, also showed that individuals with less educational attainment were more doubtful whether they wanted to receive results of genetic tests for dementia risk [24]. Information quality and participant's knowledge of the research is important factor for research participation [41]. More targeted education on genetic risk for dementia is required to ensure well informed decision in research participation that might lead to disclosure [42]. The DBRR is planning to establish a Public Involvement Panel that will contribute to the information quality and ensure that recruitment strategies are appropriate for the target audience.

Online recruitment registries are unique in their ability to prescreen and invite a large pool of motivated research volunteers, thereby efficiently recruit for observational studies and clinical trials [[7], [8], [9]]. A substantial proportion of research volunteers in online recruitment registries do not respond or show interest, reflected in response ratio (number interested / number invited) which widely varies across studies utilizing these registries from 0.19 up to 0.50 [7,8,30]. For this study response rate was slightly higher than average (0.35), which was expected as a previous study among DBRR registrants showed high interest in participating in research on the genetic risk for dementia [24].

Of the returned buccal swabs, the DNA yield was satisfactory and resulted in a 0.99 genotyping success ratio, which was also sufficient to store extra genetic material in the DBRR biobank for future research optimizing DNA donation of the registrants. The 4 % material loss of buccal swabs was very low and not due to DNA yield but mainly due to delays or losses during postal transit. Furthermore, the buccal swabs are non-invasive and user friendly for participants, as also indicated by participants response on the survey. Likewise, for the researcher, the buccal swabs are convenient in their processing as they can be stored at room temperature. So remote *APOE*-testing with buccal swabs is feasible and practical, however the preparation of testing kits requires manual preparation which is time intensive.

This study has some limitations, as the DBRR contains mainly Caucasian, higher educated individuals our sample is not representative of the general population. Previous studies suggest that *APOE*-ε4 risk may vary across different ethnic groups [15], and individuals from lower socioeconomic background are at higher risk for dementia [43]. It is important to test acceptability of genetic testing and willingness to participate in dementia research among groups that are generally underrepresented in research [44]. Another limitations was that the acceptability surveys were not completed by all participants. Motivators and barriers for enrollment and acceptance of testing might therefore not reflect the complete sample or the non-participants.

The inclusion or enrichment of *APOE*-ɛ4 carriers is important for various studies, including studies with medication and non-pharmacological therapies [[45], [46], [47], [48], [49]]. An example of a non-pharmacological lifestyle intervention trial including *APOE*-ɛ4 carriers is the PENSA study which aims to prevent cognitive decline in older adults with subjective cognitive decline [47]. The rationale to include *APOE*-ε4 carriers was based on the results from the FINGER-trial, showing that healthy lifestyle changes may be beneficial for cognition in older at-risk individuals, particularly among *APOE*-ε4 carriers [50]. Nonetheless, the exclusion of individuals without APOE-ε4 alleles may omit a significant proportion of preclinical Alzheimer's disease (AD) cases. In the A4 study, APOE-ε4 carriage was not a requirement, and approximately 40% of the amyloid-positive participants did not carry an ε4-allele [32]. Including only ε4-carriers can result in the underrepresentation of individuals with AD pathology who lack this genetic risk factor. *APOE*-ε4 carriership could also serve as an exclusion criterium. Recently, the European Medicines Agency (EMA) approved marketing authorization for Lecanemab in patients with mild cognitive impairment and mild dementia due to AD, except for *APOE*-ε4 homozygotes due to increased risk of Amyloid Related Imaging Abnormalities (ARIA) [49]. As the field is preparing for a future with personalized medicine and more prevention trials are emerging, timely identification of individuals who benefit most from disease modifying treatment or preventive measures is crucial [45,[51], [52], [53]]. To facilitate these trials, online registries might enable prescreening for other genetic risk factors or biomarkers using alternative remote collection methods [54].

Following up on our results, the DBRR will continue *APOE*-ɛ4 genotyping among self-reported cognitively normal adults in the DBRR to gather genetic prescreening data to facilitate more targeted recruitment. The DBRR has not yet used genetic information to recruit for prevention studies, hence no information on enrolment- or screen failure rates is currently available. Though, based on our results and of other online registries [21], we are confident that inviting research volunteers based on *APOE*-genotype genetically enlisted in an online registries will effectively accelerate the enrollment process for prevention trials. Future studies have the possibility to send invitations to an *APOE*-e4 enriched sample from the DBRR. The DBRR will not share genetic results with registrant, but invited registrants are informed that they might learn their genetic results when enrolling into a certain study.

Our findings show feasibility and acceptability of large-scale remote *APOE*-genotyping of cognitively-normal adults enrolled in an online registry for prescreening purposes. Our insights are encouraging for recruitment registries and prevention studies, suggesting that genetic risk prescreening, whether or not results are disclosed, is well-accepted and well-understood among cognitively normal adults. Our study validates a genetic prescreening approach previously applied in online registries in US within an online registry in The Netherlands [19,22]. Future studies will evaluate its effectiveness in accelerating recruitment and enrolment for AD prevention trials. Early screening for genetic and other risk factors, along with enrollment in AD research, can drive advancements in the field [51,52]. Online recruitment registries can play a pivotal role in these efforts to engage the public in AD research.

## Funding

The Dutch Brain Research Registry (Hersenonderzoek.nl) is supported by ZonMw‐Memorabel (project no 73,305,095,003), Alzheimer Nederland, Amsterdam Neuroscience, and Hersenstichting (Dutch Brain Foundation). This work was supported by a Crossover grant (MOCIA 17,611) of the Dutch Research Council (NWO). The MOCIA programme is a public-private partnership (see https://mocia.nl/scientific/. WF & MZ are recipients of EU-FINGERS, and MultiMemo, bothan EU Joint Programme - Neurodegenerative Disease Research (JPND) projects receiving funding from ZonMW (#733,051,102 and #JPND2022–111) and Multi-Memo, (EU Joint Program—Neurodegenerative Disease Research (JPND) Multi-MeMo grant). SK, MZ, LNCV and WF are recipients of ABOARD, which is a public-private partnership receiving funding from ZonMW (#73,305,095,007) and Health∼Holland, Topsector Life Sciences & Health (PPP-allowance; #LSHM20106). SAMS is recipient Health∼Holland, Topsector Life Sciences & Health (PPP-allowance LSHM19051, LSHM20084; LSHM22026-SGF), and ZonMW (#10,510,032,120,003, and (#7,330,502,051 and #73,305,095,008).

## CRediT authorship contribution statement

**L. Waterink:** Writing – review & editing, Writing – original draft, Visualization, Project administration, Methodology, Investigation, Formal analysis, Data curation. **S.J. van der Lee:** Writing – review & editing, Supervision, Funding acquisition. **D. Nijland:** Writing – review & editing, Project administration, Data curation. **F.I. van der Zee:** Writing – review & editing, Project administration, Data curation. **L.N.C. Visser:** Writing – review & editing. **Y.A.L. Pijnenburg:** Writing – review & editing. **S.A.M. Sikkes:** Writing – review & editing, Supervision. **W.M. van der Flier:** Writing – review & editing, Supervision. **M.D. Zwan:** Writing – review & editing, Supervision, Resources, Methodology, Funding acquisition, Conceptualization.

## Conflict of Interest Disclosure

SAMS provided consultancy services to Prothena Biosciences, Aribio and Biogen, and she is part of the Scientific Advisory Board of Cogstate. All funds are paid to the institution. WF has performed contract research for Biogen MA Inc, and Boehringer Ingelheim. WF has been an invited speaker at Biogen MAInc, Danone, Eisai, Novonordisk, Web MD Neurology (Medscape), Springer Healthcare, European Brain Council. WF is consultant to Oxford Health Policy Forum CIC, Roche, Eisai, and Biogen MA Inc. WF participated on advisory boards of Biogen MAI inc, Roche, and EliLilly. All funding is paid to her institution. WF is a member of the steering committee of PAVE,and Think Brain Health. WF was associate editor of Alzheimer, Research & Therapy in 2020/2021. WF is associate editor at Brain. MZ is site coordinator of the phase 1/2 ASPIRE-FTD clinical trial (NCT06064890) sponsored by AviadoBio. LW, SL, DN, FIZ, LNCV, YALP report no conflict of interest.

## Appendix Supplementary materials

Image, application 1
