# Supplementary material for: Feasibility and acceptability of remote APOE-genotyping among research volunteers of an online recruitment registry (The Dutch Brain Research Registry)
Source: J Prev Alzheimers Dis. 2025 Feb 24;12(5):100099. doi: 10.1016/j.tjpad.2025.100099 (PMC12183973; doi:10.1016/j.tjpad.2025.100099)
Supplement: Supplementary file 1 [file mmc1.docx]

**Supplementary Table 1.** Number of participants endorsing reasons to either (much) higher or (much) lower self-reported risk for dementia compared to the general population.

|  | **Endorsed, *n* (%)** |
| --- | --- |
| **Reasons for (much) higher risk for dementia compared to the general population** (*N* = 537) |  |
| Dementia runs in my family | 473 (88) |
| I have memory problems | 145 (27) |
| I have health problems, such as cardiovascular disease or diabetes | 89 (17) |
| I do little to prevent dementia | 19 (4) |
| Life often goes wrong for me | 15 (3) |
| **Reasons for (lower) risk of dementia compared to the general population** (*N* = 494) |  |
| Dementia does not run in my family | 308 (63) |
| I do not have health problems, such as cardiovascular disease or diabetes | 274 (56) |
| I do not have memory problems | 243 (49) |
| I do a lot to prevent dementia | 171 (35) |
| Life often goes well for me | 26 (6) |

Notes. Results are presented as the number *n* of participants (%) out of the total number participants (*N*) that responded to the question. Participants were allowed to endorse a maximum of three reasons.

**Supplementary Table 2.** Comparison of participants and non-participants.

|  | Total invited  (*n* = 9,289) |  | Participants  (*n* = 2,886) | Non- participants  (*n* = 6,403) | p-value |
| --- | --- | --- | --- | --- | --- |
| Age, (mean ±SD) | 67.3 ± 7.0 |  | 67.7 ± 6.8 | 67.1 ± 7.1 | **<.001** |
| Sex, *n* (%)  Male | 4,046 (44) |  | 1,242 (43) | 2,804 (44) | .644 |
| Female  Other | 5,231 (56)  1 (0) |  | 1,639 (57)  0 | 3,592 (56)  1 (0) |  |
| Education, *n* (%)  Vocational or less | 1,933 (21) |  | 470 (16)^▼^ | 1,463 (23)^▲^ | **<.001** |
| Higher vocational or academic | 7,341 (79) |  | 2,410 (84)^▲^ | 4,931 (77)^▼^ |  |
| Subjective memory complaints, *n* (%) | 1,769 (19) |  | 494 (17)^▼^ | 1,275 (20)^▲^ | **<.001** |
| Relative with dementia, *n* (%)  Yes | 3,491 (38) |  | 1,331 (46)^▲^ | 2,160 (34)^▼^ | **<.001** |
| No | 5,150 (55) |  | 1,413 (49)^▼^ | 3,737 (58)^▲^ |  |
| I don't know* | 489 (5) |  | 134 (5) | 355 (6) |  |

Notes: *IQR* Interquartile range. Non-participants did not respond to invitation, were not interested in participation, declined informed consent or (pre)screen failed. Participants provided consent and were sent a buccal swab to their home. *Excluded from chi-square and post-hoc analysis. Post-hoc analysis showed significant difference with: ^▲^more often reported (positively associated);^▼^less often reported (negatively associated). Number missing among total invited for sex *n* = 11; education *n* = 15; subjective memory complaints *n* = 1,264; relative with dementia *n* = 159.

**Supplementary Table 3.** Results of multivariable ordinal regression: association of demographics and prescreening questions with *APOE*-*ε*4 carriership.

|  |  | APOE-*ε*4 allele (0, 1, 2) | | |
| --- | --- | --- | --- | --- |
| Predictors |  | OR | 95% CI | p-value |
| Age |  | 0.98 | 0.97 – 1.00 | .095 |
| Sex [female] |  | 1.02 | 0.81 – 1.28 | .896 |
| Subjective memory complaints [yes] |  | 1.07 | 0.80 – 1.42 | .665 |
| Relative with dementia [yes] |  | **2.14** | **1.66 – 2.77** | **<.001** |
| Self-reported risk compared to general population  (much) smaller |  | 1.02 | 0.75 – 1.37 | .917 |
| Similar |  | ref |  |  |
| (much) higher |  | 1.30 | 0.99 – 1.72 | .058 |

Notes: OR Odds Ratio; CI confidence interval. ‘Percentage self-estimated lifetime risk’ and ‘Percentage self-estimated risk in 5 years’ were excluded for analysis due to high multicollinearity. Only complete cases are included (*n* = 1,553, *R^2^nagelkerke* = 0.642).

**
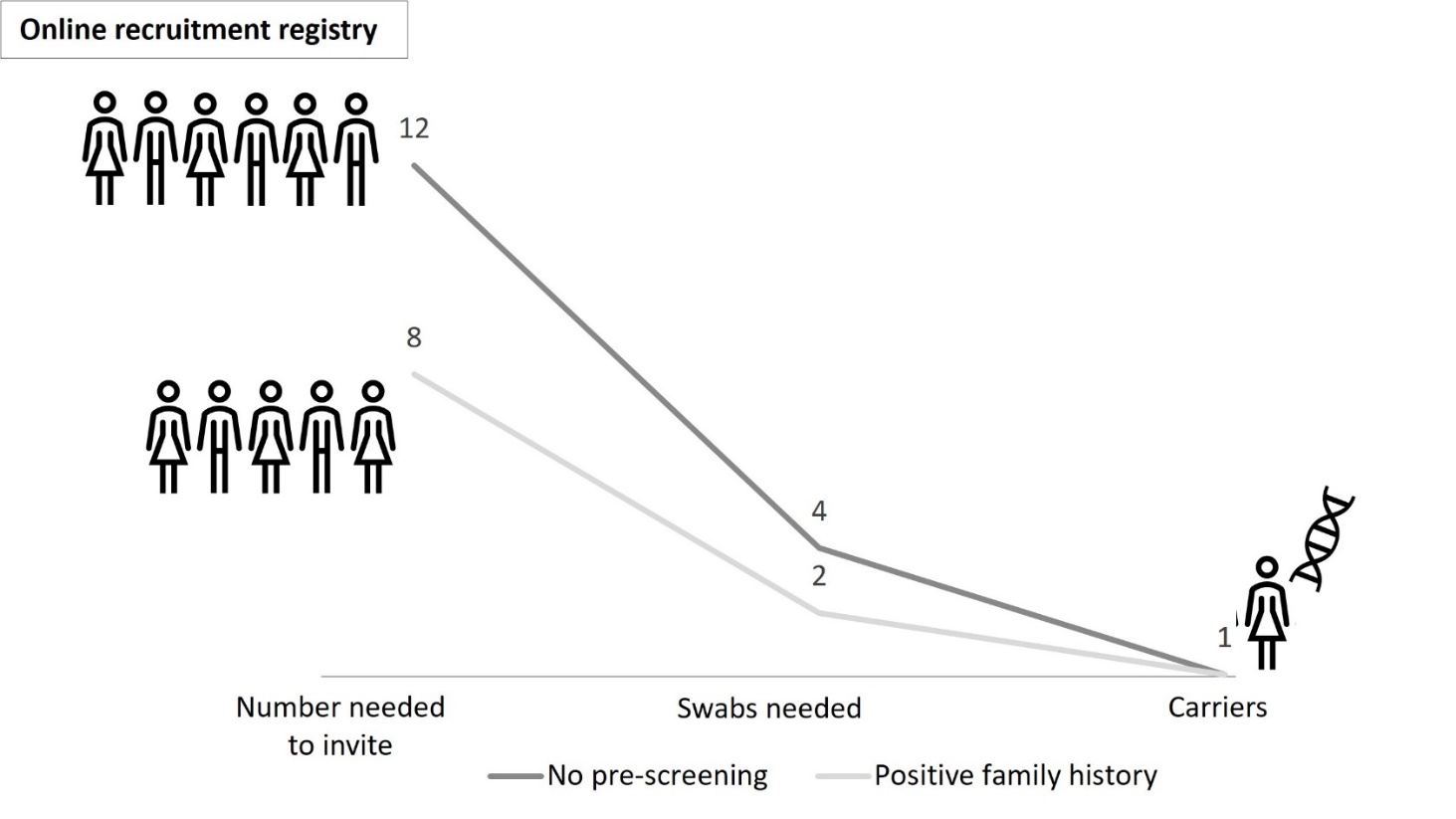
**

**Supplementary Figure 1.** Number needed to invite from the DBRR to identify one carrier with and without pre-screening for a positive family history of dementia.
